# Impact of sense of coherence and social support on disease activity in Crohn’s disease: a network analysis

**DOI:** 10.3389/fpubh.2025.1701461

**Published:** 2025-11-21

**Authors:** Xiao Han, Jinghan Liu, Jinli Bu, Zhen Wang, Zhouying Chen, Meihao Wei

**Affiliations:** Nursing Department, Sir Run Run Shaw Hospital, Zhejiang University School of Medicine, Hangzhou, China

**Keywords:** sense of coherence, social support, disease activity, Crohn’s disease, network analysis, nursing

## Abstract

**Background:**

Sense of coherence (SOC) and social support are key factors in managing chronic diseases, but their specific impact on disease activity in Crohn’s disease (CD) remains unclear. Network analysis provides a novel approach to understanding the complex relationships among psychological and clinical variables.

**Objective:**

This study aimed to use network analysis to explore the relationship between SOC and social support and disease activity in CD patients across different body weight categories, considering that body weight influences disease prognosis, nutritional status, and treatment needs.

**Methods:**

A cross-sectional study was conducted among 276 hospitalized CD patients from a tertiary hospital in Zhejiang. Participants were categorized as underweight, normal weight, or overweight based on BMI. SOC (SOC-13), social support (MSPSS), and disease activity (simplified CDAI) were assessed using validated tools. Disease activity was measured using a simplified Crohn’s Disease Activity Index (CDAI), which is an abbreviated version of the full CDAI with a reduced number of items (5 instead of 8). Partial correlation networks were constructed for each BMI group using graphical LASSO and visualized via the Fruchterman–Reingold algorithm. Centrality and bridge expected influence were calculated to identify key variables, and Network Comparison Tests were conducted across BMI groups.

**Results:**

Comprehensibility and family support emerged as central nodes in normal and overweight groups, while manageability was central in underweight patients. Bridge analyses indicated that manageability and family support linked SOC, social support, and disease activity. No significant structural differences were found among BMI groups (*p* > 0.05), suggesting consistent interrelations regardless of BMI.

**Conclusion:**

Manageability and family support were identified as central and bridging factors in CD patients, regardless of BMI. Targeted interventions to strengthen these factors may enhance disease management and patient outcomes. However, given the exploratory nature of the results, which is needed to confirm these findings.

## 1 Introduction

Crohn’s disease (CD) is a chronic, progressive inflammatory bowel disease that can affect the entire gastrointestinal tract (1). Common clinical symptoms of CD include abdominal pain, diarrhea, fever, weight loss, and fatigue, with severe cases potentially leading to gastrointestinal obstruction, perforation, and fistulas, which may require surgery (2). The etiology of CD is complex, with symptoms that may relapse, and there is currently no cure. Current pharmacological treatments mainly aim to relieve symptoms, delay disease progression, and prevent or manage complications (3). To improve quality of life, patients with CD are typically advised to modify their lifestyle and undergo long-term or even lifelong treatment. Pharmacological therapy alone is often insufficient to address the full spectrum of health needs of patients with CD, particularly the physical and psychological burdens associated with the long-term management and adaptation to a chronic illness (4–6). Therefore, the optimal treatment and rehabilitation plan for CD should not only help patients manage the disease but also focus on psychological coping strategies and self-management behaviors that may directly or indirectly alleviate symptoms, in order to maximize the improvement of overall treatment outcomes.

In 1979, medical sociologist Aaron Antonovsky introduced the Salutogenic Model, which posits that an individual’s health exists on a continuous spectrum between complete health and complete illness (7). The model advocates for individuals to proactively create and maintain health from a positive perspective. One of the core concepts of this model is the sense of coherence (SOC), which refers to an individual’s ability to promote health in the presence of external stressors (8). It explains the extent to which an individual perceives the stressors in their life as comprehensible, manageable, and meaningful. According to the Salutogenic Model, SOC is a crucial psychological factor in determining an individual’s health (9). Individuals with higher SOC are more likely to maintain a positive attitude when facing difficulties and are more capable of understanding and responding to stressors in a constructive manner (10). Research has shown that a strong SOC is positively correlated with better disease management in patients with chronic disease (11). Therefore, SOC may play a critical role in the disease control and adaptation process of patients with CD, and understanding SOC and its underlying factors provides a pathway to improving disease control outcomes in those requiring long-term treatment and management. However, to our knowledge, research on SOC in patients with CD remains scarce, and the complex relationships between the multidimensional characteristics of SOC and disease activity in CD are not yet well understood, which hinders healthcare providers from developing personalized intervention strategies based on the Salutogenic Model.

Social support refers to the array of emotional, material, informational, or companionship resources that an individual perceives or receives from their social network (12). It plays a significant role in both the SOC and the control of chronic diseases (13, 14). A robust social support network provides emotional and practical resources, making individuals’ lives more manageable and meaningful, thereby enhancing their ability to cope with life challenges. In patients with CD, higher levels of social support help alleviate psychological stress associated with the disease, thereby having a positive impact on disease control (15–17). However, previous studies have primarily focused on the impact of a single dimension of social support on disease control in patients with CD, overlooking the multifaceted nature of social support and the complex relationships between the various dimensions of social support, SOC, and disease activity in CD (15, 16). Further, individuals with a stronger SOC are better at obtaining and utilizing external resources and are more likely to actively seek and accept social support (18). The relationship between social support and SOC may be bidirectional. Therefore, it is necessary to use more appropriate statistical methods to systematically assess the multidimensional characteristics of SOC and social support, the interactions between these dimensions, and their relationship with CD disease activity, thereby providing a deeper theoretical foundation for promoting personalized management of CD.

Network analysis is a statistical method that reveals the relationships and core features among variables by analyzing their complex interactions (19, 20). Therefore, network analysis may comprehensively uncover the complex interplay between SOC, social support, and CD disease activity, identifying the core features that influence disease activity in patients with CD. In addition, body weight plays a crucial role in the course of CD. Due to the impact of the disease, patients with CD may experience nutritional absorption disorders, leading to weight loss and even malnutrition (21, 22). The varying weight statuses of patients with CD may be a reflection of their disease severity (23). Thus, we hypothesized that the patterns of the relationship network among SOC, social support, and disease activity may differ among patients with different weight statuses.

Therefore, this study aimed to employ network analysis to comprehensively explore the impact of SOC and social support on disease activity in patients with CD of different body weights, revealing the complex interrelationships among these variables. Through this study, we hope to provide a deeper theoretical foundation for developing personalized intervention strategies for CD, thereby promoting CD patients’ active creation and maintenance of health.

## 2 Materials and methods

### 2.1 Study design and setting

This cross-sectional study employed network analysis to examine the impact of SOC and social support on disease activity in CD patients across different body weight categories. All participants were recruited from the Department of Gastroenterology at a tertiary hospital in Zhejiang, where they had received a confirmed diagnosis of CD. Recruitment took place between December 2024 and May 2025. Eligible patients, who were hospitalized for Crohn’s Disease-related management (e.g., disease flare, complications, or disease monitoring), were invited to participate during their hospitalization and completed a paper-based survey containing both self-reported information and standardized questionnaires.

The inclusion criteria for this study were as follows: (1) a confirmed diagnosis of Crohn’s Disease (CD) based on the 2021 “Chinese Consensus on the Diagnosis and Treatment of Inflammatory Bowel Disease,” regardless of disease activity status (remission or active), (2) age between 18 and 45 years, and (3) the ability to understand and complete the questionnaire. Participants were excluded if they had (1) co-existing severe psychiatric disorders, or (2) had experienced a major traumatic event within the past 3 months. Participation was voluntary, with all participants providing written informed consent. The study was approved by the Ethics Committee of Sir Run Run Shaw Hospital, Zhejiang University School of Medicine (Approval No. 2024-2,610-01).

### 2.2 Participants and procedure

A total of 276 hospitalized CD patients were enrolled and completed the study questionnaires. Participants were classified into three groups based on their body mass index (BMI) as follows: underweight (BMI < 18.5), normal weight (BMI ≥ 18.5 and <24.0), and overweight (BMI ≥ 24.0). Validated instruments were used to assess social support, sense of coherence (SOC), and disease activity. Baseline evaluations included demographic characteristics, BMI, clinical symptoms, and disease-related indices. Detailed participant characteristics are presented in Table 1.

**Table 1:** Demographics of participants with Crohn’s disease based on weight.

	Total (*n* = 276)	Normal weight (*n* = 140)	Overweight (*n* = 85)	Underweight (*n* = 51)	P. overall	P. normal vs. Overweight	P. normal vs. Underweight	P. overweight vs. Underweight
Age, years (mean ± SD)	28.0 [23.0–34.0]	27.0 [22.0–34.0]	30.0 [25.0–33.0]	28.0 [23.0–34.0]	0.310	0.381	0.543	0.543
Gender					0.003	0.065	0.098	0.003
Male	218 (79.0%)	109 (77.9%)	76 (89.4%)	33 (64.7%)				
Female	58 (21.0%)	31 (22.1%)	9 (10.6%)	18 (35.3%)				
BMI	21.5 [19.2–24.5]	21.1 [19.6–22.3]	26.2 [24.8–30.1]	17.3 [16.9–18.0]	<0.001	<0.001	<0.001	<0.001
Education_level					0.468	0.755	0.755	1.000
High school or below	47 (17.0%)	20 (14.3%)	17 (20.0%)	10 (19.6%)				
University or above	229 (83.0%)	120 (85.7%)	68 (80.0%)	41 (80.4%)				
Monthly income, RMB, *n* (%)					0.036	0.439	0.095	0.023
<5,000	40 (14.5%)	19 (13.6%)	8 (9.41%)	13 (25.5%)				
5,000–10,000	123 (44.6%)	63 (45.0%)	45 (52.9%)	15 (29.4%)				
>10,000	113 (40.9%)	58 (41.4%)	32 (37.6%)	23 (45.1%)				
Disease duration, years, *n* (%)					0.656	0.977	0.731	0.731
<5	182 (65.9%)	93 (66.4%)	56 (65.9%)	33 (64.7%)				
5–10	66 (23.9%)	35 (25.0%)	21 (24.7%)	10 (19.6%)				
>10	28 (10.1%)	12 (8.57%)	8 (9.41%)	8 (15.7%)				
Biologic biosimilar therapies, *n* (%)					0.293	0.536	0.536	0.394
Yes	251 (90.9%)	127 (90.7%)	80 (94.1%)	44 (86.3%)				
No	25 (9.06%)	13 (9.29%)	5 (5.88%)	7 (13.7%)				
Immunosuppressants, *n* (%)					0.629	0.868	0.868	0.868
Yes	61 (22.1%)	34 (24.3%)	16 (18.8%)	11 (21.6%)				
No	215 (77.9%)	106 (75.7%)	69 (81.2%)	40 (78.4%)				
Surgical treatment for IBD, *n* (%)					0.917	1.000	1.000	1.000
Yes	137 (49.6%)	70 (50.0%)	43 (50.6%)	24 (47.1%)				
No	139 (50.4%)	70 (50.0%)	42 (49.4%)	27 (52.9%)				
The number of chronic diseases, *n* (%)					0.761	0.713	0.713	0.713
0	161 (58.3%)	84 (60.0%)	47 (55.3%)	30 (58.8%)				
1	101 (36.6%)	49 (35.0%)	32 (37.6%)	20 (39.2%)				
≥2	14 (5.07%)	7 (5.00%)	6 (7.06%)	1 (1.96%)				
CDAI general condition, *n* (%)					0.018	0.718	0.017	0.017
2	206 (74.6%)	111 (79.3%)	66 (77.6%)	29 (56.9%)				
3	60 (21.7%)	25 (17.9%)	17 (20.0%)	18 (35.3%)				
4	5 (1.81%)	2 (1.43%)	0 (0.00%)	3 (5.88%)				
5	5 (1.81%)	2 (1.43%)	2 (2.35%)	1 (1.96%)				
CDAI abdominal pain, *n* (%)					0.192	0.840	0.185	0.185
2	162 (59.1%)	82 (59.4%)	53 (62.4%)	27 (52.9%)				
3	99 (36.1%)	51 (37.0%)	30 (35.3%)	18 (35.3%)				
4	13 (4.74%)	5 (3.62%)	2 (2.35%)	6 (11.8%)				
CDAI diarrhea, *n* (%)	1.00 [1.00–2.00]	1.00 [1.00–2.00]	1.00 [1.00–2.00]	1.00 [1.00–2.50]	0.618	0.663	0.663	0.663
CDAI abdominal mass, *n* (%)					0.155	0.210	0.459	0.188
2	251 (91.3%)	128 (91.4%)	79 (94.0%)	44 (86.3%)				
3	15 (5.45%)	6 (4.29%)	5 (5.95%)	4 (7.84%)				
4	9 (3.27%)	6 (4.29%)	0 (0.00%)	3 (5.88%)				
CDAI comorbidities, *n* (%)	1.00 [1.00–2.00]	1.00 [1.00–2.00]	1.00 [1.00–2.00]	1.00 [1.00–2.00]	0.945	0.900	0.900	0.900
CDAI scores, *n* (%)	5.00 [3.00–6.00]	5.00 [3.00–6.00]	4.00 [3.00–6.00]	5.00 [4.00–7.00]	0.204	0.317	0.317	0.242
Family support, *n* (%)	23.0 [18.0–26.0]	22.0 [18.0–25.2]	24.0 [18.0–26.0]	23.0 [18.5–26.0]	0.578	0.716	0.908	0.716
Friend support, *n* (%)	20.0 [16.0–24.0]	20.0 [17.0–24.0]	20.0 [16.0–24.0]	20.0 [16.0–24.0]	0.823	0.898	0.898	0.898
Other support, *n* (%)	20.0 [16.0–24.0]	20.0 [16.8–24.0]	20.0 [16.0–25.0]	19.0 [16.0–23.0]	0.531	0.936	0.551	0.551
Comprehensibility, *n* (%)	20.0 [15.0–23.0]	20.0 [16.0–23.0]	19.0 [14.0–22.0]	20.0 [16.5–22.0]	0.415	0.431	0.972	0.431
Manageability, *n* (%)	18.0 [12.0–21.0]	18.5 [12.0–21.0]	17.0 [11.0–20.0]	19.0 [13.0–20.0]	0.366	0.499	0.815	0.499
Meaningfulness, *n* (%)	17.0 [15.0–19.0]	17.0 [15.0–19.0]	17.0 [15.0–18.0]	17.0 [15.5–19.0]	0.978	0.984	0.984	0.984
Total social support, *n* (%)	62.0 [52.0–72.0]	62.0 [54.0–72.0]	62.0 [50.0–73.0]	63.0 [51.5–71.5]	0.949	0.896	0.896	0.896

The continuous variables were presented as median (interquartile range, IQR), and the categorical variables were shown as number and percentages. BMI, body mass index; SD, standard deviation.

### 2.3 Multidimensional scale of perceived social support (MSPSS)

Social support was assessed using the Multidimensional Scale of Perceived Social Support (MSPSS), which was developed by Zimet et al. (24). It measures social support from three sources: family, friends, and significant others. The instrument contains 12 items, each rated on a 7-point Likert scale (1 = “very strongly disagree” to 7 = “very strongly agree”). Total scores are obtained by summing all items (range: 12–84), with higher scores indicating greater perceived social support. The MSPSS has been verified in China (25). The Cronbach’s *α* coefficient in this study is 0.95. In this study, scores for each source of support were calculated separately to investigate their potential differential associations with disease activity.

### 2.4 Simplified Crohn’s disease activity index (CDAI)

Disease activity was evaluated using the simplified Crohn’s Disease Activity Index (CDAI) (26). This widely used clinical scoring system quantifies CD severity to guide therapeutic decision-making. The CDAI incorporates five components: (1) abdominal pain: rated from 0 (none) to 3 (severe); (2) diarrhea: scored with 1 point for each occurrence of loose stools per day; (3) general wellbeing: rated from 0 (very good) to 4 (very poor); (4) abdominal mass: rated from 0 (none) to 3 (confirmed with tenderness) and (5) extraintestinal manifestations/complications: one point is assigned for each of the following conditions: joint pain, iritis, erythema nodosum, pyoderma gangrenosum, oral aphthous ulcers, anal fissures, new fistulas, and abscesses (1 point per condition). The total score for the simplified CDAI is calculated by summing the scores for each of the five domains. The disease activity levels are then categorized as follows: Remission: Total score ≤4; Mild activity: Total score 5–7; Moderate activity: Total score 8–16; Severe activity: Total score >16. Due to its ease of use and comprehensive assessment capability, the CDAI is widely employed in both clinical practice and research (27–30).

### 2.5 Sense of coherence (SOC) assessment

The 13-item Sense of Coherence (SOC-13) scale, developed by Antonovsky et al. and revised to a Chinese version by Bao et al., was used to assess SOC (8, 31). The SOC-13 comprises three dimensions: comprehensibility, manageability, and meaningfulness. Each item is scored on a 7-point Likert scale ranging from 1 (“very often”) to 7 (“never”), yielding a total score between 13 and 91, with higher scores indicating a stronger SOC. The Cronbach’s *α* coefficient in this study is 0.82.

### 2.6 Statistical analysis

Continuous variables were summarized as mean ± standard deviation (SD) if normally distributed, or as median and interquartile range (IQR) if non-normally distributed. Categorical variables were presented as frequencies and percentages. Baseline differences between groups were analyzed using chi-square tests (*χ*^2^), one-way analysis of variance (ANOVA), or Kruskal–Wallis tests, as appropriate. Analyses were stratified by weight status to explore potential differences across BMI categories. Missing data were handled using multiple imputation (MICE). All statistical analyses were performed in R version 4.4.3, with a significance threshold of *p* < 0.05.

#### 2.6.1 Network estimation

Partial correlation networks among social support dimensions, SOC, and disease activity were estimated separately for each weight category using the graphical least absolute shrinkage and selection operator (glasso). Network estimation and visualization were performed using the qgraph package in R software. In these networks, nodes represent study variables and edges represent conditional dependencies between variables after adjusting for all others. Non-parametric normalization (NPN) transformation was not applied, as the data were sufficiently normalized for network analysis.

To prevent spurious associations and optimize network parsimony, the extended Bayesian information criterion (EBIC) was applied for model selection (32). The Fruchterman–Reingold algorithm was used for network layout, positioning strongly connected nodes closer together (33). Separate networks were estimated for normal-weight, overweight, and underweight patients to assess weight-specific patterns. Edge thickness and color saturation indicate partial correlation strength; positive associations are shown in blue, and negative associations in red.

#### 2.6.2 Network inference

Centrality metrics-strength, closeness, and betweenness-were computed to quantify node importance (34). Strength reflects the sum of absolute edge weights directly connected to a node; closeness captures the inverse of the average shortest path length to other nodes; betweenness measures the frequency with which a node lies on the shortest path between other nodes.

Expected influence (EI), which incorporates both positive and negative edge weights, was also calculated (35). Bootstrapping (*n* = 2,000) using the bootnet package estimated 95% confidence intervals for edge weights and calculated the correlation stability coefficient (CS-coefficient) for centrality indices. A CS > 0.25 indicates interpretable stability, and CS > 0.50 reflects good stability. Case-dropping bootstrapping was also performed to test robustness, repeatedly re-estimating networks after removing random subsets of cases. The random seed was set to 12,345 for reproducibility.

#### 2.6.3 Network comparison

To examine weight-related differences in network structure, the Network Comparison Test (NCT) was used (36). This two-tailed permutation test compares network structure invariance, global strength (sum of absolute edge weights), and individual edge weight differences between groups. One thousand permutations were performed for pairwise comparisons between the overweight and underweight groups. Higher global strength indicates tighter overall connectivity, which may be positively associated with disease activity.

## 3 Results

### 3.1 Sample characteristics

Baseline characteristics of participants, stratified by BMI category, are presented in Table 1. A total of 276 eligible patients with Crohn’s disease were ultimately included in the network analysis. The median age of the overall sample was 28 years (IQR: 23.0–34.0), with 218 participants (79.0%) identified as male and 58 (21.0%) as female. The sample had a higher proportion of male participants (79.0%) compared to the average gender distribution reported for CD in China.

The median BMI for the overweight group was 26.2 (IQR: 24.8–30.1), for the underweight group was 17.3 (IQR: 16.9–18.0), and for the normal weight group was 21.1 (IQR: 19.6–22.3). Compared to the overweight and normal-weight groups, the underweight group had a higher proportion of females, as well as a greater prevalence of both low-income and high-income individuals, and notably, poorer general health status and higher disease activity scores (all *p* values < 0.05).

### 3.2 Network analysis results

#### 3.2.1 General network structure

**Figure 1:**
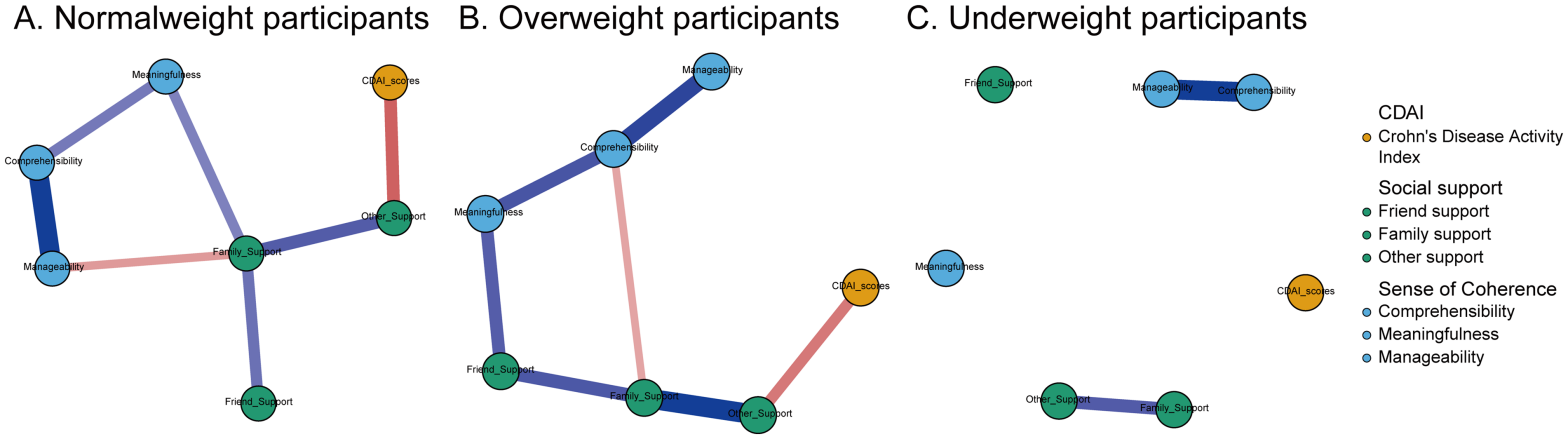
Network analysis of the relationships among social support, sense of coherence, and disease activity in participants with normal weight (**A**, *n* = 140), overweight (**B**, *n* = 85), and underweight (**C**, *n* = 51). Edge thickness reflects the strength of the association between variables. Blue edges indicate positive connections, while red edges indicate negative connections between nodes.

In network analysis, each variable (e.g., SOC dimensions, social support types, disease activity indicators) is represented as a node, and their statistical associations are represented as edges. A central node refers to a variable with the highest influence within the network, commonly assessed using strength centrality, which quantifies the sum of the absolute weights of its direct connections to other nodes. A node with high centrality is considered crucial for maintaining the integrity and function of the network. Conversely, a peripheral node has few and/or weak connections with other nodes, indicating it plays a less influential or isolated role within the network. The network structures comprising dimensions of perceived social support, sense of coherence (SOC), and disease activity are illustrated in Figure 1. Blue edges indicate positive associations, while red edges indicate negative associations.

In the normal weight group, comprehensibility emerged as the central node of the network, showing strong positive connections with both manageability and meaningfulness (components of SOC). Family support appeared as another key node, positively linked to friend support and other support, and negatively associated with manageability. Moreover, other support was indirectly associated with overall disease activity scores. Friend support was the most peripheral node in this network.

A similar pattern was observed in the overweight group, where comprehensibility remained the most central node, positively associated with both manageability and meaningfulness, but negatively associated with family support. Family support also played a central role, connected to friend support, other support, and manageability, and indirectly linked to disease activity via other support.

In contrast, among underweight patients, no strong associations were identified between SOC, social support dimensions, and disease activity, indicating a potentially fragmented or unstable psychological network in this subgroup.

#### 3.2.2 Stability of centrality indices and edge precision

**Figure 2:**
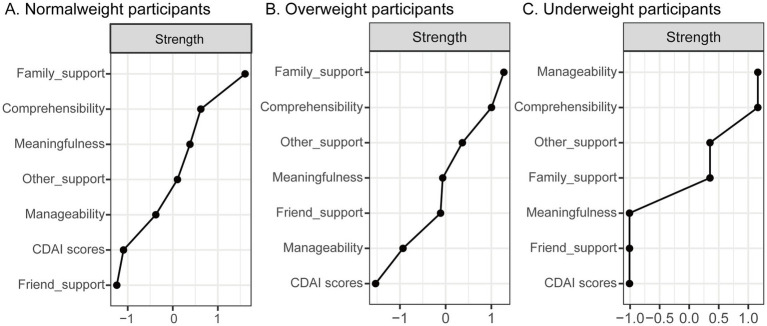
Standardized z-scores (strength centrality) of variables in patients with Crohn’s disease, categorized by normal weight (**A**, *n* = 140), overweight (**B**, *n* = 85), and underweight (**C**, *n* = 51). Higher standardized strength values indicate that a variable is a central node in the overall structure and function of the network.

**Figure 3:**
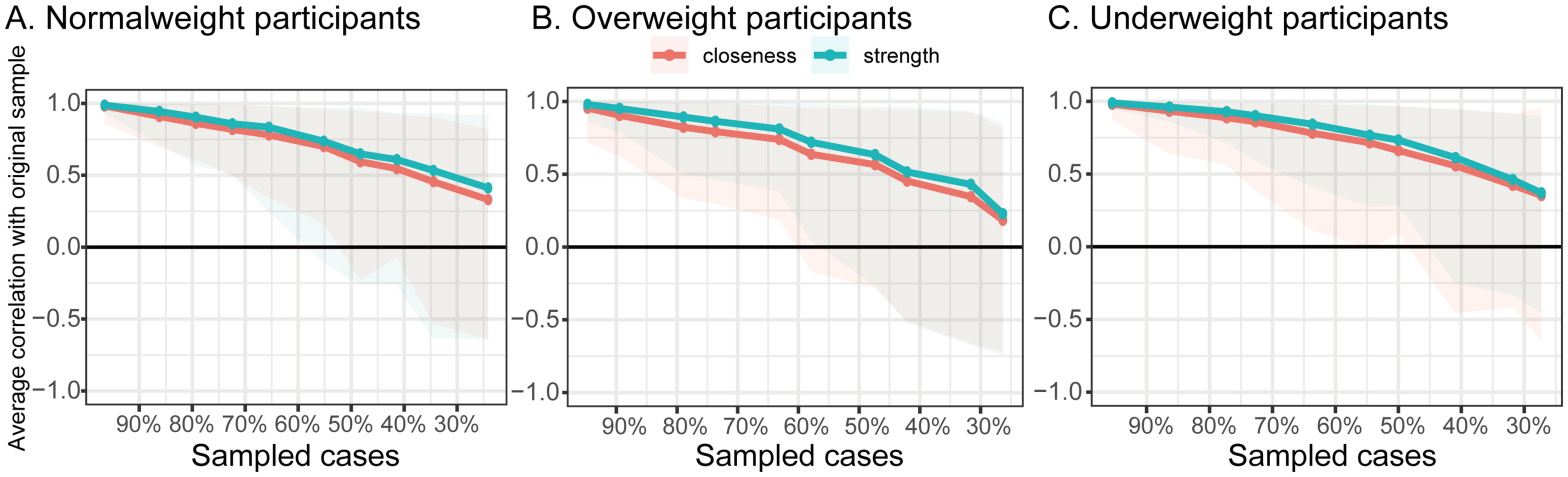
Estimated 95% bootstrap confidence intervals for the edges in the network constructed based on social support, sense of coherence, and disease activity in Crohn’s disease patients. The results are shown for the normal weight group (**A**, *n* = 140), overweight group (**B**, *n* = 85), and underweight group (**C**, *n* = 51).

**Figure 4:**
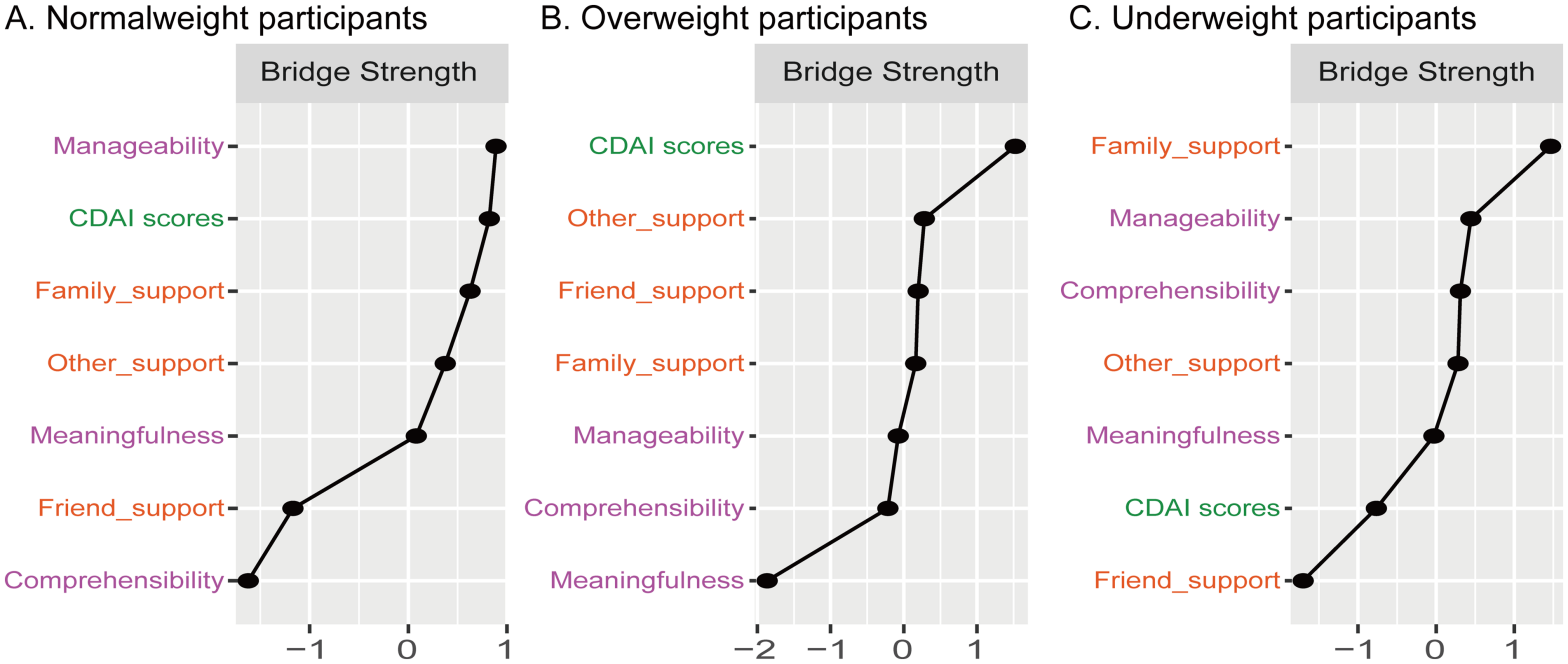
Standardized bridging centrality strength scores of nodes in the network constructed based on social support, sense of coherence, and disease activity in patients with Crohn’s disease (*n* = 276). Results are shown for the normal weight group (**A**, *n* = 140), overweight group (**B**, *n* = 85), and underweight group (**C**, *n* = 51). Higher values indicate a stronger mediating role of a node in bridging different communities (modules).

Standardized strength centrality values across the three BMI subgroups are shown in Figure 2. In both the normal weight and overweight groups (Figures 2A,B), the core variables were family support and comprehensibility. However, in the underweight group (Figure 2C), manageability and comprehensibility emerged as the most central nodes (Figure 3). The correlation stability (CS) coefficients of node strength were 0.21, 0.11, and 0.27 for the normal weight, overweight, and underweight groups, respectively (Figure 4). According to established guidelines (37), a CS coefficient above 0.25 is considered the minimum threshold for interpretable results, while values above 0.50 are required to indicate moderate stability. Therefore, although the underweight group yielded the highest CS value among the three subgroups, it still falls short of moderate stability. These relatively low stability coefficients suggest that centrality estimates may be sample-sensitive and should be interpreted with caution.

#### 3.2.3 Edge and node centrality stability and difference testing

**Figure 5:**
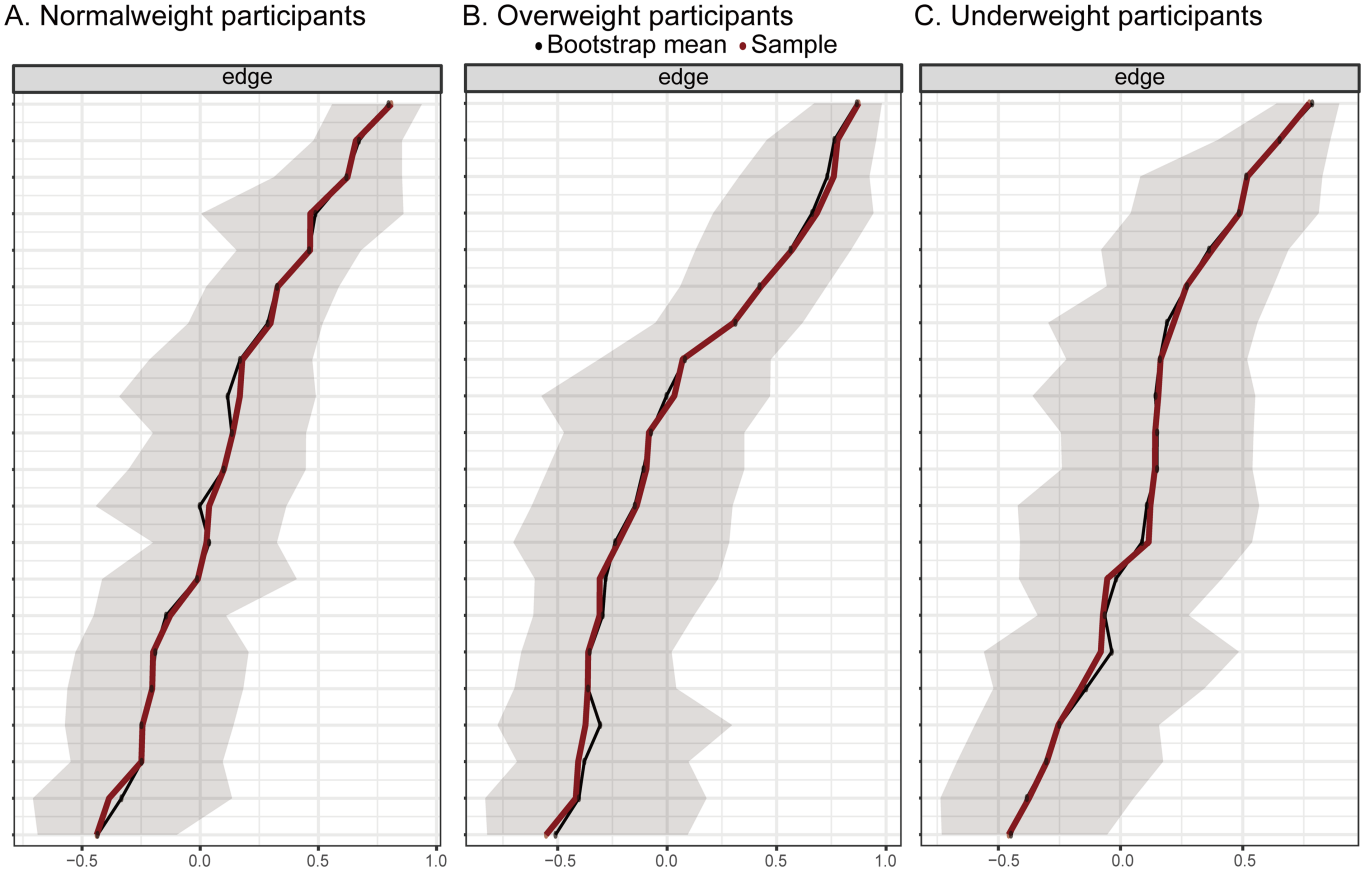
Stability of centrality metrics, including strength and closeness, in Crohn’s disease patients: normal weight participants (**A**, *n* = 140), overweight group (**B**, *n* = 85), and underweight participants (**C**, *n* = 51). The *y*-axis represents the mean correlation coefficient between the centrality scores of the original sample and those of the subsample as the sample size decreases. The line indicates the mean value, and the shaded area represents the 95% confidence interval.

Bootstrapped 95% confidence intervals for edge weights across all subgroups showed no substantial widening (Figure 5), suggesting acceptable estimation stability. However, the observed overlap of these confidence intervals across BMI groups indicates that the differences in edge weights between subgroups may not be statistically significant.

As shown in Figure 4, manageability (a SOC component) and family support exhibited the highest bridge expected influence values in the normal weight group, highlighting their critical role in linking clusters of variables from social support, SOC, and disease activity domains. This suggests that manageability and family support are not only central to the psychological network of CD patients but may also serve as key leverage points for interventions aimed at modulating disease activity and improving patient wellbeing.

In the overweight group (Figure 4), manageability and other support showed the highest bridge centrality, implying their importance in maintaining network connectivity and potentially influencing cross-domain dynamics.

**Figure 6:**
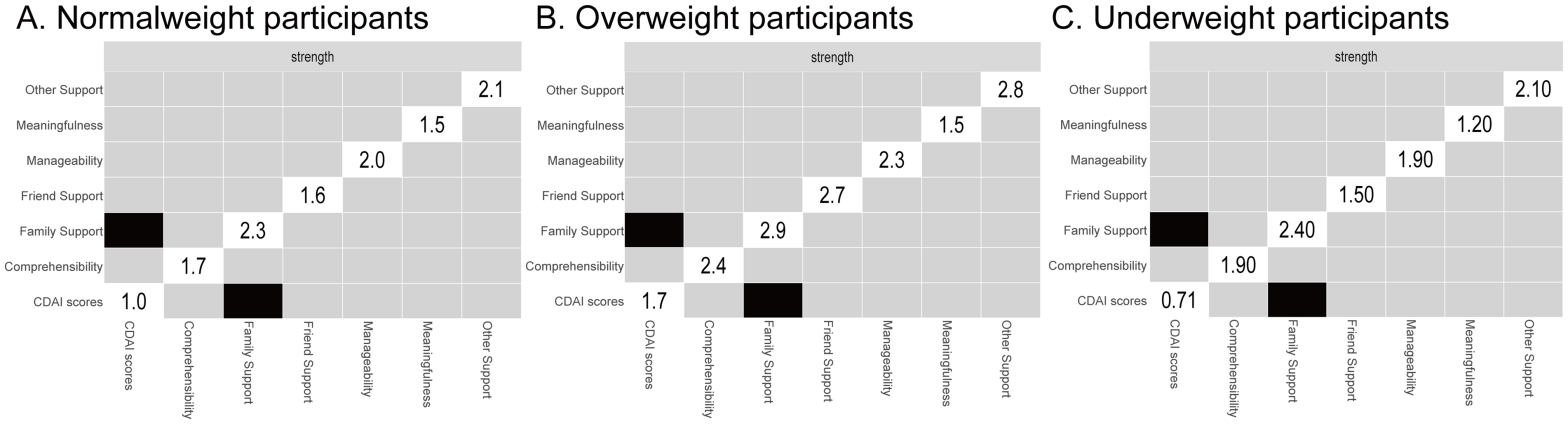
Differential bootstrap tests (*α* = 0.05) for the edges between nodes in the network constructed based on social support, sense of coherence, and disease activity in Crohn’s disease patients. The results are shown for the normal weight group (**A**, *n* = 140), overweight group (**B**, *n* = 85), and underweight group (**C**, *n* = 51). Gray boxes indicate no significant strength differences between nodes, while black boxes indicate significant strength differences. The strength values for each node are displayed along the diagonal.

In addition, as shown in Figure 6, when performing significance testing for the differences in centrality strength across the various subgroups, it was found that the centrality strength of family support was significantly higher than that of other nodes, indicating that this node has more and stronger connections with SOC and disease activity.

#### 3.2.4 Network comparison

Results of the omnibus test for network structure invariance and the Network Comparison Test (NCT) for global strength invariance revealed no statistically significant differences across BMI-based subgroups (normal weight, overweight, underweight) (all *p* values > 0.05). These findings suggest that despite minor variations in specific node-level connections, the overall architecture and global connectivity strength of the networks remained consistent across body weight categories (Table 2). Despite the absence of global structural differences, the variations in node centrality and bridge roles across BMI subgroups should be interpreted with caution. These findings are exploratory in nature and may reflect limitations such as insufficient sample size or statistical needed to validate the stability and robustness of the observed network structures.

**Table 2:** Comparison of global network features and structural characteristics across weight groups.

	Normal weight participants (*n* = 140)	Overweight participants (*n* = 85)	Underweight participants (*n* = 51)	Network1 vs. 2	Network1 vs. 3	Network 2 vs. 3
Global strength	5.99	5.83	2.04			
Top 5 most central nodes (strength centrality)	Family support (1.46)	Family support (1.26)	Comprehensibility (0.63)			
	Comprehensibility (1.09)	Comprehensibility (1.17)	Manageability (0.63)			
	Manageability (1.00)	Other support (0.95)	Family Support (0.39)			
	Other support (0.89)	Meaningfulness (0.81)	Other Support (0.39)			
	Meaningfulness (0.71)	Friend support (0.80)	CDAI scores (0)			
NCT results						
Omnibus test of network structure invariance *p*-value				0.95	0.871	0.356
Global strength invariance test *p*-value				0.376	0.238	0.059

NCT, Network Comparison Test; Network 1, normal weight participants; Network 2, overweight participants; Network 3, underweight participants.

## 4 Discussion

This study used network analysis to examine the complex associations between SOC, social support, and disease activity in patients with CD of different body weights, and three main findings were made. First, in normal-weight patients with CD, comprehensibility and family support are the nodes with the highest centrality in the network. Manageability and family support are the nodes with the highest bridging strength in the network, thus connecting SOC, social support, and disease activity more strongly within the network. As shown in Figure 6, when performing significance testing for the differences in centrality strength across the various subgroups, it was found that the centrality strength of family support was significantly higher than that of other nodes, indicating that this node has more and stronger connections with SOC and disease activity. Second, in overweight patients with CD, comprehensibility and family support are the nodes with the highest centrality in the network. Manageability and other support are the nodes with the highest bridging strength in the network, playing a more important role in maintaining network connectivity. Third, in underweight patients with CD, no significant connection was found between SOC, social support, and disease activity. The results of this study provide new insights into the interrelationship between SOC, social support, and disease activity in patients with CD of different body weights. However, it is worth noting that although networks of different body weights have distinct characteristics, the comparison of networks did not reveal significant differences. This may be due to the large sample size differences among the three groups, particularly the small sample size in the underweight group. Nevertheless, to the best of our knowledge, this is the first study to investigate the complex relationships between SOC, social support, and disease activity in CD patients with different body weights.

Previous studies have shown that SOC, as an internal resource, helps patients with chronic diseases such as diabetes, cancer, and heart disease to understand, cope with, and manage stress in life, potentially slowing disease progression and improving the quality of life of patients (11, 38, 39). However, there is a current lack of attention to SOC in patients with CD. Social support provides emotional comfort, informational support, and practical assistance to patients with chronic disease, and is considered a key factor influencing the disease burden and prognosis (40, 41). Several studies have highlighted the significance of social support for the health of patients with CD (15, 16). Although these studies emphasize the role of high levels of social support in disease control in patients with CD, they have not used network analysis methods to explore the complex relationships between these factors, nor have they considered potential differences in these associations among patients with different body weights. This study is the first to explore the relationship between SOC, social support, and disease activity through network analysis, considering the potential impact of body weight on this relationship, offering new insights and perspectives for the development of personalized treatment plans for patients with CD.

The study results reveal that comprehensibility and family support are central elements in the network of normal-weight and overweight patients with CD, suggesting that these two factors play a critical role in the disease activity of CD. On the one hand, patients with higher comprehensibility are more likely to effectively understand the concepts, symptoms, treatment options, and potential progression of the disease, thus being more likely to understand and adhere to treatment plans, and consequently manage the disease more easily (42). On the other hand, family support also plays an important role in the disease management of patients with CD. Patients with CD typically require long-term lifestyle adjustments, and the reduction in quality of life due to disease symptoms leads to a need for greater physical and emotional support (43). Support from family members, particularly in dietary management, medication adherence, emotional healing, and daily life, helps patients establish a healthy lifestyle, relieve stress, and enhance their confidence in coping with the disease, thereby reducing the negative impacts of the disease (44). Thus, the strong correlation between family support for life and emotional aspects plays a vital role in helping patients with CD cope with the disease.

In addition, in normal-weight CD patients, manageability and family support are the nodes with the highest bridging strength in the network, indicating that they play a bridging role in connecting the relationships between SOC, social support, and disease activity. Manageability refers to the extent to which patients believe they have sufficient resources and abilities to cope with their disease (8). Even if patients can understand their disease (high level of comprehensibility), they may still be unable to manage the disease effectively if they lack appropriate capabilities and resources (45). Therefore, manageability plays a transmitting role between the dimensions of SOC and also connects the interaction between the patient’s SOC and social support. Moreover, in normal-weight patients with CD, family support is both a central node and one of the nodes with the highest bridging strength between SOC and social support, playing a crucial role in disease control by providing practical and emotional support to patients. However, for overweight patients with CD, the high bridging strength of manageability and other support suggests that these patients may require more help from external resources, such as assistance from leaders and colleagues, professional medical support, or other social support systems, when managing their disease. Although family support still plays the role of a central node, overweight CD patients often need to make more efforts in weight management and disease control (46, 47). Therefore, they may be more likely to rely on broader social support to compensate for the lack of self-management. Notably, in both normal-weight and overweight patients with CD, we found that family support is negatively correlated with manageability and comprehensibility. This may suggest that high levels of family support may, in certain cases, excessively protect patients, making them more reliant on their family for understanding and coping with the disease rather than on themselves.

However, compared to normal-weight and overweight patients with CD, underweight patients did not show a significant relationship between SOC, social support, and disease activity, which may be related to their poorer health condition. Underweight patients suffer from malnutrition, which often indicates disease deterioration. As a result, they have a poorer quality of life, more fragile emotions, and lack sufficient physical strength and energy to cope with the challenges posed by the disease (48, 49). This may make them more dependent on medical interventions rather than proactive self-management when facing the disease. In addition, patients with more severe CD are more likely to have psychological issues such as anxiety and depression, which may make it harder for them to perceive and accept effective social support relationships (50, 51). Therefore, poor disease control and malnutrition may weaken the association between SOC, social support, and disease activity.

The differences in network characteristics among patients with CD of different body weights highlight the importance of developing personalized support strategies in CD disease management. In normal-weight and overweight patients with CD, comprehensibility and family support play a crucial role in disease control. Therefore, disease management should first strengthen patients’ disease awareness education and improve their disease manageability, while also helping family members provide appropriate family support through education and training, especially in areas such as lifestyle management and emotional support. However, it is important to note that family members should not overprotect the patient. Overweight patients with CD may face more complex weight control and disease management issues. Thus, for overweight patients with CD, in addition to strengthening family support, attention should also be given to the utilization of other social support systems, such as medical support, workplace support, and other community resources. Furthermore, underweight patients with CD may lack effective SOC and the ability to perceive and accept social support due to issues like malnutrition, disease progression, and negative emotions, making basic health education insufficient for effective intervention. Therefore, interventions should focus on providing emotional and psychological support to help patients enhance their emotional regulation and stress coping abilities, while also increasing medical intervention and nutritional support (52).

Although this study provides new insights into the complex relationship between SOC, social support, and disease activity in patients with CD of different body weights, there are still some limitations. First, this study used a cross-sectional design, which cannot reveal the causal relationship between SOC, social support, and disease activity. Therefore, future studies should consider using a longitudinal design to more clearly track the dynamic changes and causal effects between these factors. Second, the sample in this study mainly comes from a specific region, which may introduce regional bias. Therefore, the generalizability of the findings needs to be further validated in other regions or larger sample populations. Third, the items related to SOC and social support and some CDAI items are self-reported by patients, which may introduce recall bias. Fourth, although this study used network analysis methods, it only considered the relationship between SOC, social support, and disease activity. Future research could explore more factors, such as patients’ mental health, disease self-management abilities, and the availability of healthcare resources, to comprehensively assess the impact of different factors on disease activity in patients with CD. Fifth, our study sample was characterized by a high proportion of male participants (79.0%). This gender imbalance, potentially attributable to regional referral patterns or healthcare-seeking behaviors specific to the tertiary hospital in Zhejiang from which the sample was drawn, may limit the generalizability of our findings to female CD populations or other healthcare settings. Sixth, it is important to acknowledge that the CDAI includes body weight as one of its components, which introduces an inherent dependency between BMI and disease activity. This dependency could partly explain the higher disease activity observed in the underweight group. Given the role of body weight in the CDAI, lower BMI in underweight patients could result in an overestimation of disease activity, contributing to their poorer health status. Therefore, future studies should consider this dependency and investigate how nutritional status and body weight impact disease activity more explicitly in underweight patients with Crohn’s disease. Furthermore, while bootstrapping analyses were conducted to estimate the precision of edge weights and the robustness of the network structure, the overlapping confidence intervals across BMI groups imply that differences in edge weights between subgroups may not be statistically meaningful. Therefore, interpretations regarding between-group comparisons should be made with caution, and future studies with larger samples may help to clarify these subgroup-specific network differences. Finally, the limited sample size, especially for underweight patients, may have constrained the statistical power of the NCT to identify significant global network differences, even though distinct node-level patterns were observed. Therefore, the network relationships identified in this study, particularly for underweight patients, should be considered preliminary and require further investigation in larger cohorts. Future studies with larger sample sizes are needed to further investigate the network associations and potential differences among SOC, social support, and disease activity.

## 5 Conclusion

This study explores the network of SOC, social support, and disease activity in patients with CD of different body weights, clarifying the interrelationships among these factors. It is important to interpret the lack of significant structural differences in the NCT across BMI groups with caution, as this finding is exploratory and could be influenced by the sample size and statistical power, rather than unequivocally demonstrating structural stability or theoretical robustness. In the networks of normal-weight and overweight patients with CD, both comprehensibility and family support emerged as central nodes, while manageability and family support, as well as manageability and other support, served as bridging nodes in each respective network. These stable patterns point to potentially important roles of these nodes in the interplay between psychological and clinical variables. In underweight patients with CD, no significant networks were identified, which may be related to poorer health condition and malnutrition. Future longitudinal studies are warranted to examine whether enhancing these key psychological and social support factors could contribute to improved disease outcomes. Our findings underscore the need to consider personalized support strategies for CD patients, tailored by body weight and psychosocial profiles.

## Data Availability

The raw data supporting the conclusions of this article will be made available by the authors, without undue reservation.
